# Global research activity on mathematical modeling of transmission and control of 23 selected infectious disease outbreak

**DOI:** 10.1186/s12992-022-00803-x

**Published:** 2022-01-21

**Authors:** Waleed M. Sweileh

**Affiliations:** Department of Physiology, Pharmacology/Toxicology, Division of Biomedical Sciences, College of Medicine and Health Sciences, An-Najah National University, Nablus, Palestine

**Keywords:** Mathematical modeling, Infectious diseases, transmission, Prevention, Research activity

## Abstract

**Background:**

Mathematical analysis and modeling allow policymakers to understand and predict the dynamics of an infectious disease under several different scenarios. The current study aimed to analyze global research activity on mathematical modeling of transmission and control of several infectious diseases with a known history of serious outbreaks.

**Methods:**

Relevant publications were retrieved using a comprehensive validated search query. The database used was SciVerse Scopus. Indicators related to evolution, growth of publications, infectious diseases encountered, key players, citations, and international research collaboration were presented.

**Results:**

The search strategy found 5606. The growth of publications started in 1967 and showed a sharp rise in 2020 and 2021. The retrieved articles received relatively high citations (h-index = 158). Despite being multidisciplinary, *Plos One* journal made the highest contribution to the field. The main findings of the study are summarized as follows: (a) COVID-19 had a strong impact on the number of publications in the field, specifically during the years 2020 and 2021; (b) research in the field was published in a wide range of journals, mainly those in the field of infectious diseases and mathematical sciences; (c) research in the field was mainly published by scholars in the United States and the United Kingdom; (d) international research collaboration between active countries and less developed countries was poor; (e) research activity relied on research groups with a large number of researchers per group indicative of good author-author collaboration; (f) HIV/AIDS, coronavirus disease, influenza, and malaria were the most frequently researched diseases; (g) recently published articles on COVID-19 received the highest number of citations; and (h) researchers in the Eastern Mediterranian and South-East Asian regions made the least contribution to the retrieved articles.

**Conclusion:**

Mathematical modeling is gaining popularity as a tool for understanding the dynamics of infectious diseases. The application of mathematical modeling on new emerging infectious disease outbreaks is a priority. Research collaboration with less developed countries in the field of mathematical epidemiology is needed and should be prioritized and funded.

## Background

Infectious diseases have been a constant threat to humanity [1]. This threat has been intensified in the past few decades due to the emergence and re-emergence of several fatal infectious diseases [2]. In 2019, the World Health Organization (WHO) has released the top ten health threats for mankind for the coming decade. The list included several items related to infectious diseases such as Ebola, malaria, Zika, and Acquired Immunodeficiency Syndrome (AIDS) [3]. In late 2019, the Coronavirus disease (COVID-19) was identified in Wuhan, China, and has spread worldwide, leading to an ongoing pandemic killing hundreds of thousands of people [4]. Experts in the field of infectious diseases expect that global warming and climatic changes will bear dynamic changes in the transmission and epidemiology of several serious infectious diseases [5, 6].

The prevention and control of an infectious disease depend on the detailed understanding of the pathophysiology and mode of transmission of that disease. Scientists, use mathematics and certain host-pathogen or host-vector interaction data to develop a mathematical model that can predict how infectious diseases spread and the number of people expected to get the infection under certain conditions. In other words, a mathematical model is a mathematical equation that describes changes in the system with time. A mathematical model can be used to forecast the impact of certain measures, such as social distancing, wearing masks, avoiding mass gatherings, and vaccination on the number of people who will get the infection. Therefore, mathematical modeling is a tool for the analysis of the dynamics of infectious diseases and potential control strategies.

The use of mathematical models in epidemiology started as early as 1760 by Daniel Bernoulli who was working on the epidemiology of Smallpox in England [7]. The modern mathematical modeling started with Ross in his paper published in 1911 in which he investigated the dynamics of malaria transmission [8]. Following up with Ross, Kermack and McKendrick founded the deterministic compartmental epidemic modeling suggesting that the probability of infection of a susceptible individual is analogous to the number of his contacts with infected individuals and the rate at which susceptible individuals become infected is given by kSI where S and I represent population densities of susceptible and infected people, respectively while k is a constant [9–11]. A key finding in mathematical modeling is the basic reproduction number “Ro” which is the average number of secondary infections caused by an average infective individual. If the basic reproduction number (Ro) is less than one, this means that the disease dies out and greater than one means that there is a possibility that an epidemic will occur [12]. The deterministic compartmental model created by Kermack and McKendric was preceded by the probabilistic model (stochastic epidemic modeling) developed by Enko in 1889 to describe the epidemic of measles in discrete-time [13].

The emergence and re-emergence of several global infectious diseases [14], including the current global epidemic of COVID-19, have led to the overwhelming practice of mathematical modeling which requires the collaboration of scientists in the field of mathematical sciences and public health. Furthermore, the COVID-19 pandemic led to the extensive use of mathematical models to study disease transmission dynamics and to evaluate the number needed to treat or vaccinate to prevent epidemics [15].

To update the young generation of scientists on global research activity on mathematical modeling of infectious diseases, the current study was undertaken. Scientific publications are considered the thoughtful contribution of scholars on a specific topic. Therefore, the number of publications contributed by each country is a source of prestige for that country and a source of attraction for international students for training, education, research, and work. Analysis of research activity on mathematical modeling sheds light on the extent of international research collaborations that need to be prioritized by funding agencies to increase the research capacities of professionals and academics in less developed countries. For a specific country, analysis of literature on mathematical modeling provides examples of how other countries developed successful measures to control and eradicate specific infectious diseases such as malaria. Countries need to learn from the experience of other countries regarding evidence-based control measures implemented to fight infectious diseases. Analysis of publications on mathematical modeling of infectious diseases and the growth of publications with time encourage academics in the field of public health and epidemiology to introduce the concept into medical curricula especially when teaching courses in the field of global health and health security. Analysis of research activity on a particular topic helps scholars identify topics of great interest and topics in need of future research efforts.

Analysis of publications on a certain topic cannot be achieved manually, especially when the number of publications is large. It is usually carried out using bibliometric methodology in which a scientific database is selected to retrieve and analyze the relevant publications [16–21]. In the past, bibliometric studies focused on research growth, citation analysis, key players in a specific field, and international research collaboration [16, 20, 22–25]. In recent years, bibliometrics was advanced by the introduction of visualization techniques applied to understand the evolving trends of research topics in various fields [21]. No bibliometric studies on the mathematical modeling of infectious diseases have been published yet. Therefore, the current study was undertaken to provide scholars with an updated analysis of published literature on mathematical modeling for the transmission and control of infectious diseases. There is a vast number of infectious diseases and pathogens that could cause harm to humans. To sharpen the analysis, the current study investigated the literature on mathematical modeling for selected infectious diseases with a known history of regional or global outbreaks [26–28].

## Methods

### Database used

The SciVerse Scopus database was selected to carry out the current study for several reasons. First, the large number and language diversity of journals indexed in Scopus compared to other databases such as PubMed or Web of Science. The number of journals indexed in Scopus is almost equal to the number of journals indexed in PubMed and Web of Science combined. Second, all journals indexed in PubMed are also indexed in Scopus, therefore PubMed is 100% inclusive in Scopus. Third, Scopus has journals in all subject areas including medicine, health, mathematics, computer, and social sciences [29]. Fourth, Scopus allows the researchers to develop complex and comprehensive search queries using different Boolean operators. Finally, Scopus allows the researcher to export and analyze the retrieved data. This includes statistical analysis and mapping.

### Research strategy

The research strategy was developed based on an extensive review of literature on the topic [30–34]. The research strategy was based on three arms.

1. In the first arm, terms and phrases related to mathematical modeling were used in the title/abstract search as follows: title-abstract (“math* model*” or “simulation model*” or “transmission model*” or (math* and model*)).
2. The second arm included terms related to transmission and control and was used in the title/abstract as follows: title-abstract (transmission, spread, propagation, dissemination, intervention, vaccin*, immuni*, control, “screening program”, preventive, isolation, regulation, mitigat*, prophylaxis, prophylactic, hygiene, testing, detection, diagnosis, eradication, elimination, policy, protection, protective, measure*, assessment).
3. The third arm of the research strategy included terms/phrases related to the selected infectious diseases and were used in the title search: title(tubercul*, syphilis, malaria/Plasmodium falciparum/Anopheles, dengue, ebola, influenza/"h1n1”/"h5n1”/flu/"avian *flu*”/"swine *flu*”, zika, “middle east respiratory syndrome”/MERS/ coronavirus/"mers-cov”/“severe acute respiratory syndrome”/“SARS/COVID-19, hantavirus, “hiv”/"human immunodef* virus”/"acquired immun*”, pneumonia, rabies, measles, cholera, smallpox, polio*, plague, or chikungunya, cholera, yellow fever).

In the research strategy, the asterisk was used as a wild card while the quotation marks were used to limit the search for the exact term/phrase.

The three arms were combined and filtered using the following exclusions and limitations. Documents published in the following journals were excluded: hydrology, physics, “ieee”, agricult*, ecolog*, plant, or botany. Also documents having the following terms in the title/abstract were excluded: plant, botany, “in vitro”. The research strategy was limited by: (1) the study period from 1900 to 2021 and (2) research or review articles as well as conference papers published in peer-reviewed journals. No language restriction was imposed. Documents on plant/agricultural infectious diseases were excluded because the main focus of the current study was on infectious diseases/ affecting humans.

### Validation

The adopted research strategy was validated for the absence of false-positive results by reviewing the title and abstract of documents with even numbers (2, 4, 6, 8, etc) up to number 200. Detected false-positive results were excluded by adjusting the research strategy. For example, certain false-positive results on mathematical modeling of infectious diseases in plants were found and excluded. The fine-tuning and exclusion of false-positive results continued until the screened random results were free of any false-positive results. The validation of the research strategy for the absence of false-negative (missing results) was accomplished by investigating the research productivity of five active authors in the field and comparing the numbers obtained with those obtained through the research strategy using the Spearman correlation test. The results showed a significant and strong correlation (*p* < 0.001; *r* = 0.96) suggestive of a high validity of the research strategy. This approach of validation was adopted by Sweileh et al. [25].

### Data export

The retrieved data were exported to Microsoft Excel for analysis and tabulation. The exported data included the annual growth of publications, types of the retrieved documents, subject areas of the retrieved documents, countries, institutions, journals, funding sponsors, and authors involved in publishing the retrieved documents. The retrieved data was also exported to the VOSviewer, a free online program [35] for mapping purposes. The map produced by VOSviewer has several characteristics: the node size which is proportional to the occurrence of the item, the node color which signifies close relation with nodes having similar color, and the thickness of connecting lines between nodes in the research collaboration map which signifies the strength of research collaboration. Citation analysis was presented as the total number of citations, mean number of citations per document, and *h*-index (Hirsch – index) which was used as a measure of scientific impact [36].

### Bibliometric indicators and data presentation

The retrieved documents were analyzed for specific bibliometric indicators and presented either as tables or graphs or maps. Active countries, journals, institutions, and authors were presented as the top ten active ones in a table format. The annual growth of publications was presented as a linear figure using the SPSS program (Statistical Program for Social Sciences, version 21). International research collaboration and bibliographic coupling of active journals were presented as network visualization maps using VOSviewer. Bibliographic coupling occurs when two articles reference a common third article in their bibliographies and it is an indication that the two articles discuss a related subject [37]. Bibliographic coupling is computed and visualized using VOSviewer program. Finally, the contribution of each world region to the retrieved data was carried out using the WHO world region classification; the region of the Americas, the European region, the Western Pacific region, the South-East Asian region, the Eastern Mediterranean region, and the African region.

## Results

### General characteristics of the retrieved articles

The research strategy retrieved 5606 articles. The retrieved articles included research articles (*n* = 5222; 93.2%), review articles (*n* = 322; 5.7%), and conference papers (*n* = 62; 1.1%). Tendifferent languages were encountered in the retrieved articles with English being the dominant (*n* = 5450; 97.2%). The remaining languages included Chinese, Spanish, Russian, French, Italian, German, and Japanese. The retrieved documents were published in journals categorized within one of the following five major different subject areas, with the potential of overlap: medicine (*n* = 2597, 46.3%), mathematics (*n* = 1687; 30.1%), immunology/microbiology (*n* = 1206; 21.5%), biochemistry/genetics (*n* = 1131; 20.2%), and agricultural/biological science (*n* = 911; 14.0%).

### Evolution and growth of publications

**Fig. 1:**
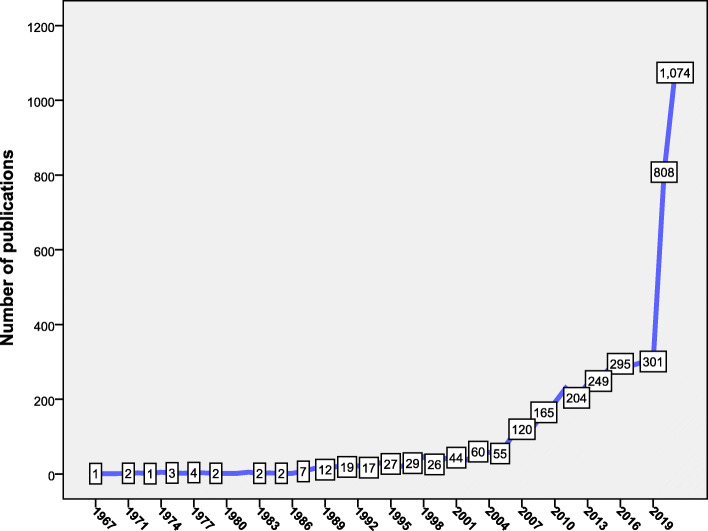
Annual growth of publications on mathematical modeling for the transmission and control of the selected infectious diseases

Figure 1 shows the evolution and annual growth of publications. The growth pattern can be organized into four stages: the first stage (1967–1986) in which the number of publications was constant and less than five articles per year; the second stage (1987–2005) in which the number of publications increased slowly; the third stage (2006–2019) in which the number of publications showed a notable increase; and finally the fourth stage (2020–2021) in which the number of publications showed a sudden upward increase. The number of articles published during the third stage (*n* = 1882, 33.6%) represented one-third of the total retrieved articles.

### Top ten active journals

The retrieved articles were published in 1071 different journals. The *Plos One* journal ranked first (*n* = 337; 6.0%) followed by the *Malaria Journal* (*n* = 119, 2.1%) and the *Journal of Theoretical Biology* (*n* = 110; 2.0%). The top ten active journals were listed in Table 1. Only two of the journals in the active list were in the field of infectious diseases (*Malaria* and *AIDS* journals) while the remaining journals were in the field of mathematical sciences or multidisciplinary. All journals in the active list have a relatively high impact factor. Of all the active journals, articles published in *Proceedings Of The National Academy Of Sciences Of The United States Of America* (PNAS) received the highest number of citations per article followed by those published in *AIDS* journal.

**Table 1:** Top ten active journals in publishing articles on mathematical modeling for the transmission and control of the selected infectious diseases

Rank	Journal Name	Frequency (%) ***N*** = 5606	Impact Factor*	Number of citations per article
1	*Plos One*	337 (6.0)	3.240	19.8
2	*Malaria Journal*	119 (2.1)	2.885	32.4
3	*Journal Of Theoretical Biology*	110 (2.0)	2.691	31.1
4	*AIDS*	107 (1.9)	4.166	51.0
5	*Chaos Solitons and Fractals*	96 (1.7)	5.944	28.2
6	*Scientific Reports*	95 (1.7)	4.13	12.7
7	*Bulletin Of Mathematical Biology*	88 (1.6)	1.758	35.4
8	*Mathematical Biosciences And Engineering*	84 (1.5)	2.08	13.9
9	*Mathematical Biosciences*	81 (1.4)	2.144	44.7
10	PNAS**	76 (1.4)	11.2	119.4

*Impact Factor for each journal was obtained from the latest Journal Citation Report published by Clarivate

**Proceedings of the National Academy of Sciences of the United States Of America

**Fig. 2:**
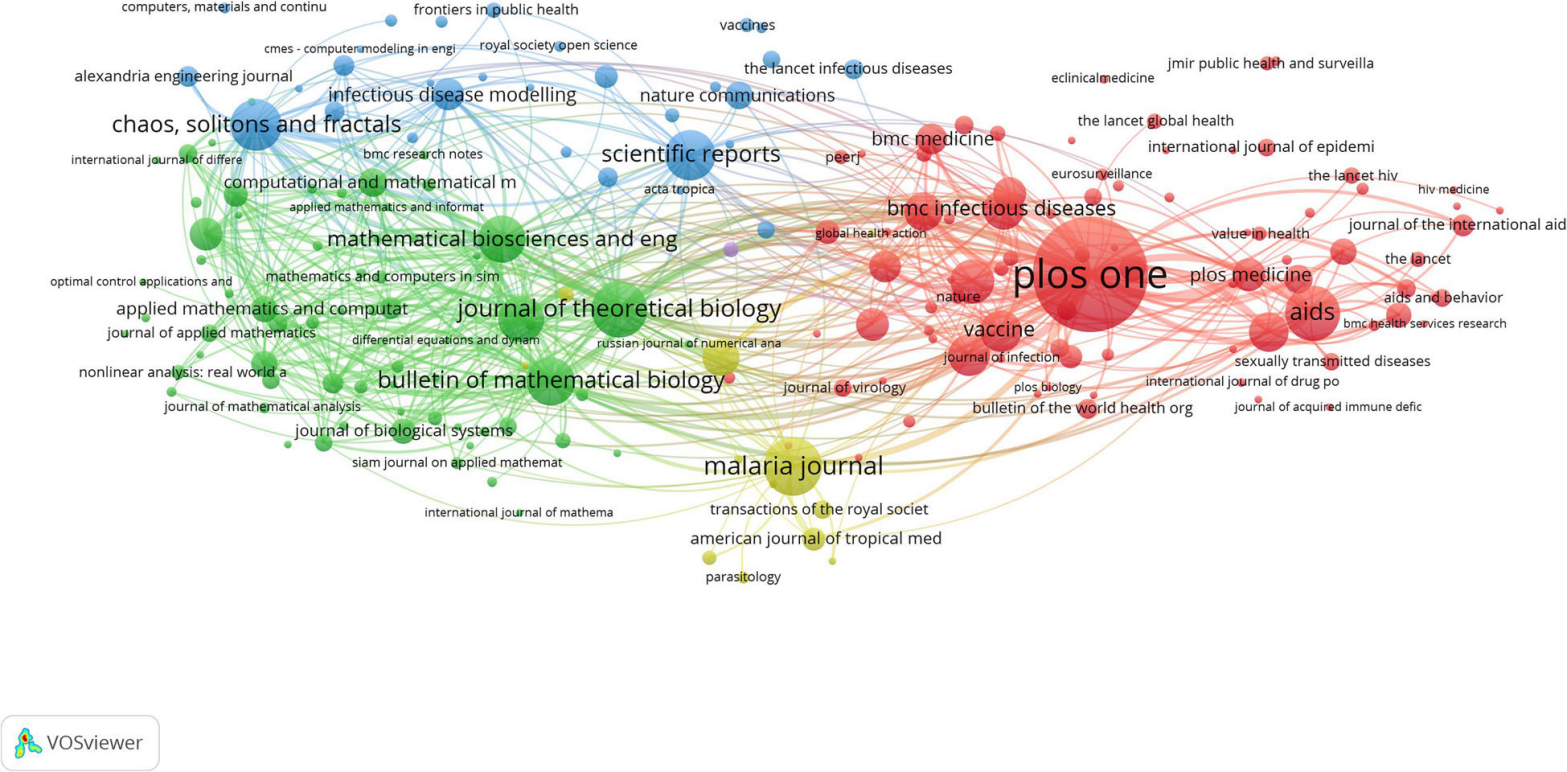
Network visualization map of bibliometric coupling of journals with a minimum contribution of 5 articles. Each cluster represented journals with a similar citation pattern. In the red cluster, most journals were bibliographically coupled with *PloS One* and *AIDS* journals while in the green cluster, most journals were bibliographically coupled with the *Bulletin of Mathematical Biology* journal, *Journal of Theoretical Biology*, and *Mathematical Biosciences* journals. In the yellow cluster, most journals were bibliographically coupled with *Malaria* and *Plos Neglected tropical diseases* journals. In the blue cluster, most journals were bibliographically coupled with *Scientific Reports* and *Choas, Soliton, and Fractals* journals

Figure 2 is a network visualization map of bibliometric coupling of journals with a minimum contribution of five articles. The map shows 196 journals distributed into two large and two small clusters. Each cluster represented journals with a similar citation pattern. In the red cluster, most articles published in medical/health journals were bibliographically coupled with *PloS One* journal while in the red cluster, most articles published in journals in the mathematical sciences field were bibliographically coupled with the *Bulletin of Mathematical Biology/ Mathematical Biosciences/ Mathematical Biosciences and Engineering/ Journal of Theoretical Biology journals.* In the small yellow cluster, articles published in journals in the field of neglected tropical diseases were bibliographically coupled with the *Malaria* journal. The fourth (blue) cluster included several journals in different (medical and non-medical) fields bibliographically coupled with the *Scientific Report* and *Chaos Solitons and Fractals* journals.

### Top ten active countries

Researchers from 156 different countries participated in the retrieved documents. Of the 156 countries, 79 (50.6%) contributed to more than 10 articles each. The USA led with 2181 (38.9%) followed by the UK (*n* = 1048; 18.7%). Table 2 lists the top ten active countries. When the research activity was standardized, India ranked first followed South Africa and China. When active countries were compared based on the mean number of citations per article, articles contributed by the UK ranked first followed by those from Switzerland and the USA. The geographical analysis of the retrieved articles based on WHO regions indicated that the region of the Americas had the highest contribution (*n* = 2671; 47.6%) followed by the European region (*n* = 2171; 38.7%) and the Western Pacific region (*n* = 1098; 19.6%). The Eastern Mediterranean region had the least contribution (*n* = 376; 6.7%) followed by the South-East Asian region (*n* = 575; 10.3%) and the African region (*n* = 855; 15.3%).

**Table 2:** Top ten active countries in publishing articles on mathematical modeling for the transmission and control of the selected infectious diseases

Rank	Country	Frequency (%) ***N*** = 5606	*Number of publications (*10^**−3**^) per GDP per capita	Number of articles without international authors (%)	Number of articles with international authors (%)	Number of citations per article
1	United States	2181 (38.9)	34.3	955 (43.8)	1226 (56.2)	38.4
2	United Kingdom	1048 (18.7)	26.0	263 (25.1)	785 (74.9)	46.4
3	China	468 (8.3)	46.8	212 (45.3)	256 (54.7)	20.1
4	Canada	413 (7.4)	9.6	123 (29.8)	290 (70.2)	27.4
5	South Africa	361 (6.4)	70.8	60 (16.6)	301 (83.4)	30.8
6	India	322 (5.7)	169.5	194 (60.2)	128 (39.8)	15.3
7	Switzerland	299 (5.3)	3.5	51 (17.1)	248 (82.9)	44.0
8	Australia	292 (5.2)	5.6	120 (41.1)	172 (58.9)	23.1
9	France	269 (4.8)	7.0	50 (18.6)	219 (81.4)	29.4
10	Netherlands	210 (3.7)	16.7	52 (24.8)	158 (75.2)	34.4

*GDP per capita: Gross Domestic Product (nominal) per capita obtained from World Bank data 2020

### International research collaboration

**Fig. 3:**
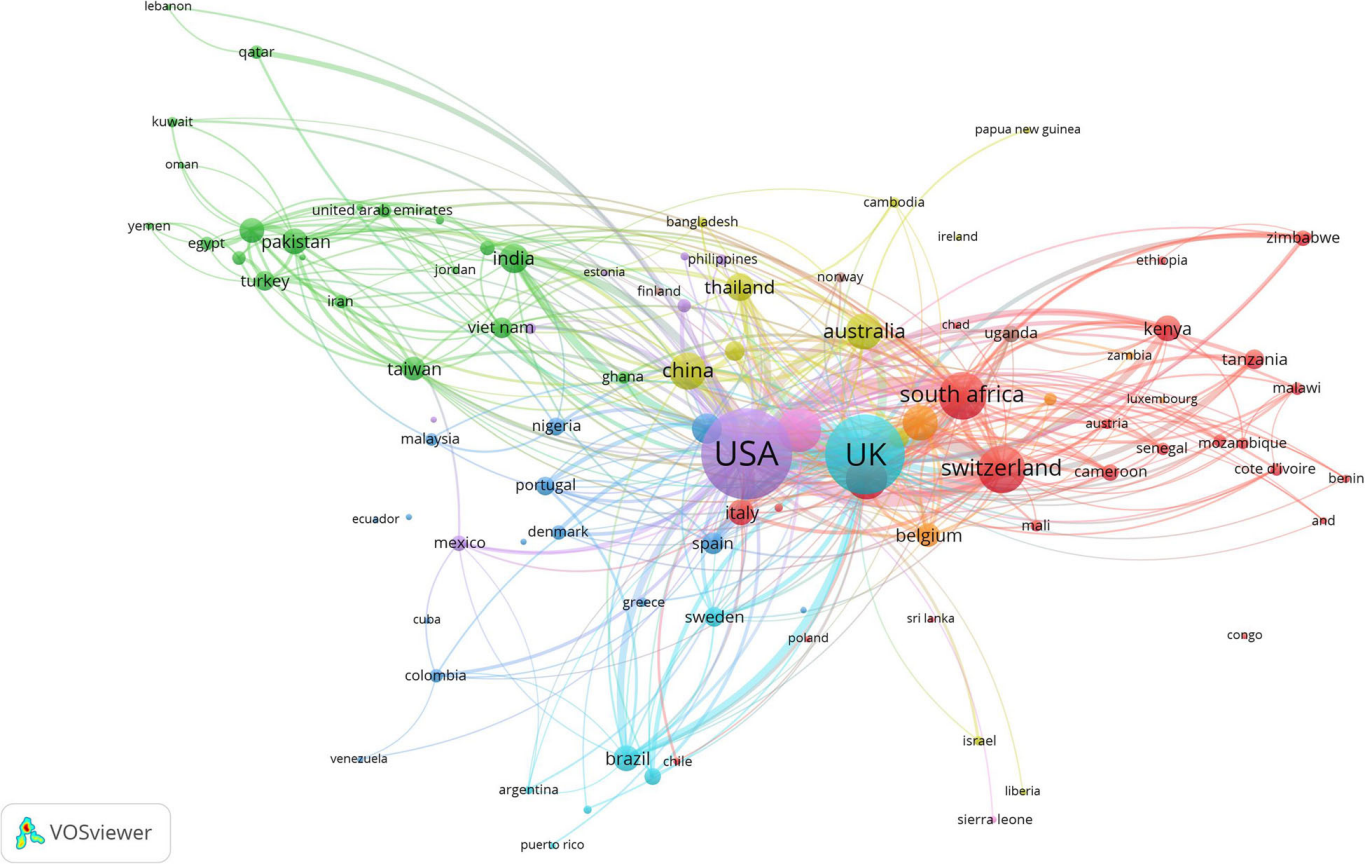
Network visualization map of international research collaboration. Countries with close distances and thick connecting lines have strong research collaboration. Countries in the periphery with thin connecting lines with countries in the center have poor international research collaboration

Table 2 also shows the extent of international research collaboration for each country in the active list. Articles contributed by South Africa had the highest percentage of international authors followed by those from Switzerland and France. Figure 3 is a network visualization map of research collaboration among countries (*n* = 96) with a minimum contribution of 5 articles. The map was characterized by (1) the presence of many countries at the margin of the map and far away from the center, and (2) the presence of many thin connecting lines between countries at the periphery and countries in the center. These characteristics indicated poor international research collaboration in the field especially between countries in the center of the map and those at the periphery. Countries in Europe, Northern America, Australia, and China had thick connecting lines among each other and were located nearby indicative of strong research collaboration.

### Authorship analysis

**Fig. 4:**
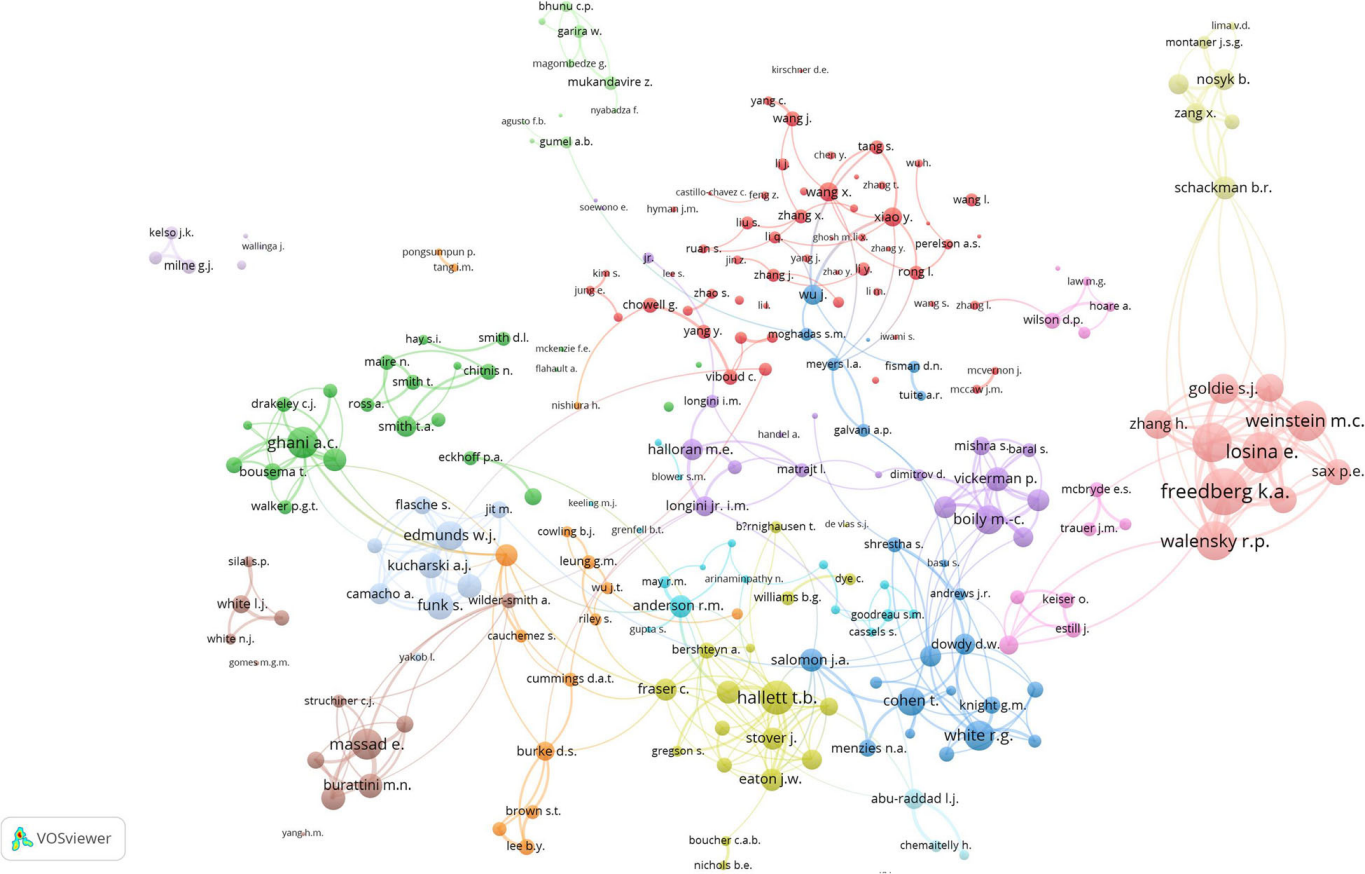
Collaborative research networks between researchers with five or more publications (*n* = 242). The map has 15 clusters with high inter and intra-cluster research collaboration as identified by the number of researchers per cluster and the relative distances between the clusters

A total of 26,908 authors participated in publishing the retrieved articles, a mean of 4.8 authors per article. Table 3 shows the top ten active authors. Hallett, T.B. (*n* = 54;) appeared to be the most active author in this field. Figure 4 shows collaborative connections among authors (*n* = 242) who published at least 5 articles in the retrieved literature. The map shows the existence of 15 research networks, 14 of the networks had a minimum of five researchers per cluster. The largest collaboration network (red cluster) represents an international research group composed of 41 scholars affiliated to institutions in the USA, UK, and China. The largest cluster included one of the top 10 active authors, specifically, Pereleson, A.S. The second-largest collaboration network (green) had 23 researchers including Ghani, A. C, one of the top ten active authors. Only one cluster (light blue) included less than five researchers in the network.

**Table 3:** Top ten active authors in publishing articles on mathematical modeling for the transmission and control of the selected infectious diseases

Rank*	Author	Frequency	% ***N*** = 5606	Country affiliation as shown in Scopus personal profile
1	Hallett, T.B.	54	1.0	UK
2	Perelson, A.S.	47	0.8	USA
3	White, R.G.	46	0.8	UK
4	Anderson, R.M.	44	0.8	UK
5	Ghani, A.C.	42	0.7	UK
5	Gumel, A.B.	42	0.7	USA
7	Garnett, G.P.	41	0.7	USA
8	Longini, I.M.	40	0.7	USA
8	Vickerman, P.	40	0.7	UK
10	Boily, M.C.	38	0.7	UK

*In the ranking system equal values were given the same rank and one rank is skipped

### Top ten active institutions

The top ten active institutions were listed in Table 4. The *Imperial College London* ranked first with 368 (6.6%) articles followed by the *London School of Hygiene and Tropical Medicine* with 335 (6.0%) articles. The top ten active institutions were mainly based in the UK and the USA. The list included six academic and four research institutions.

**Table 4:** Top ten active institutions in publishing articles on mathematical modeling for the transmission and control of the selected infectious diseases

Rank	Institution	Frequency (%) ***N*** = 5606	Country affiliation
1	*Imperial College London*	368 (6.6)	UK
2	*London School of Hygiene & Tropical Medicine*	335 (6.0)	UK
3	*Harvard University*	234 (4.2)	USA
4	*University of Oxford*	189 (3.4)	UK
5	*Centers for Disease Control and Prevention*	160 (2.6)	USA
6	*Medical Research Council*	158 (2.8)	UK
7	*Johns Hopkins University*	154 (2.7)	USA
8	*University of Washington*	146 (2.9)	USA
9	*Organisation Mondiale de la Santé*	124 (2.2)	WHO
10	*National Institutes of Health NIH*	114 (2.0)	USA

### Most frequent infectious diseases encountered in the retrieved literature

**Fig. 5:**
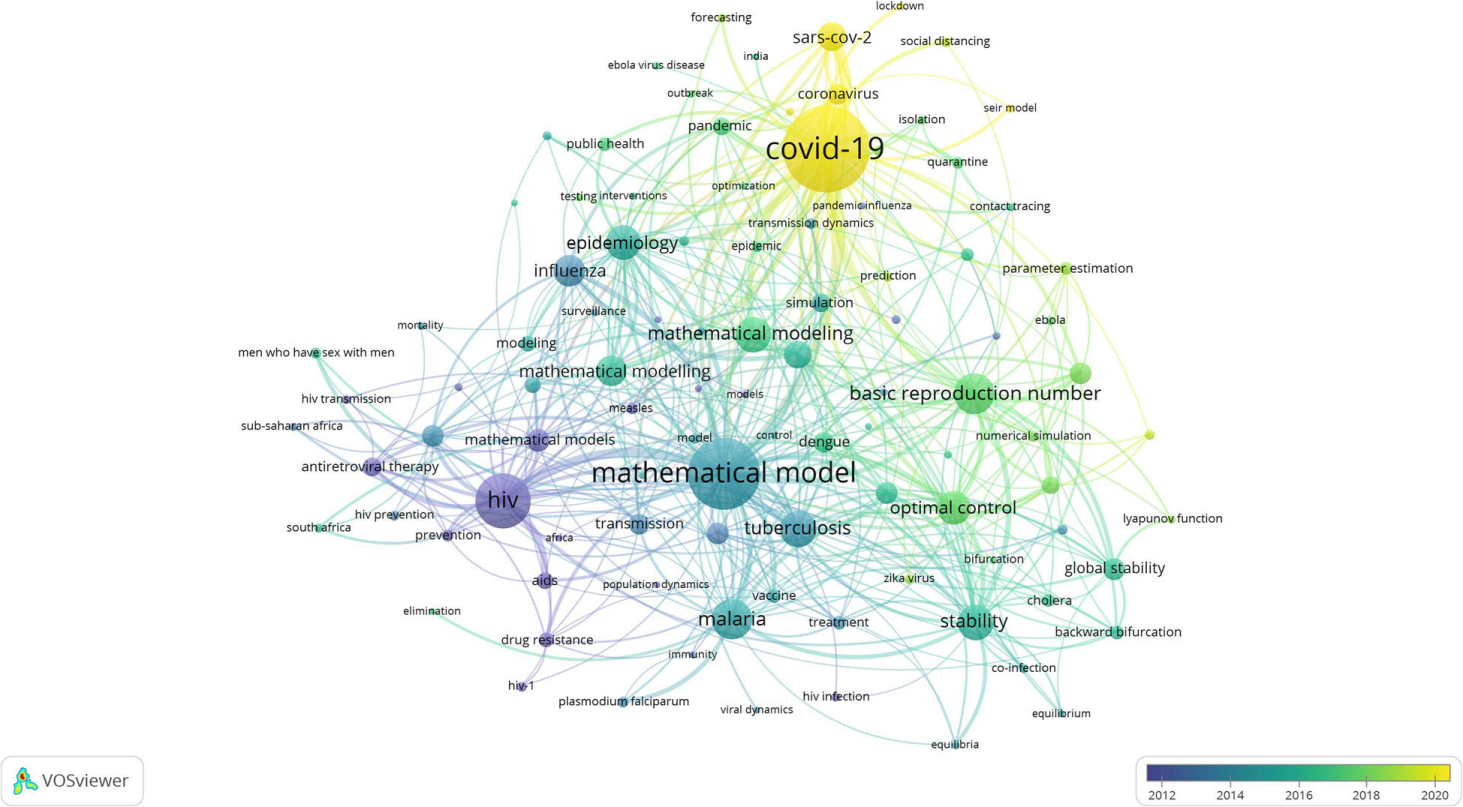
Network visualization map of the most 100 frequently encountered author keywords. The color-coding shows the time of publication or the keywords with yellow color representing the most recent author keywords

Table 5 lists the most frequently encountered infections in the retrieved articles. The HIV/AIDS (1537; 27.4%) was the most commonly encountered infection followed by coronaviruses (1316; 23.5%) and influenza (*n* = 692; 12.3%%). Figure 5 is a co-occurrence network map of the most frequent 100 author keywords in the retrieved articles. In the map, the larger the circle, the higher the occurrence of the keyword. The shorter the distance between any two items, the stronger the relation between them. Colors were used to identify the relative time of publication of the keywords with the yellow color representing the most recently published keywords. Mathematical modeling of COVID-19 was the most recent as identified by the yellow color in the map.

**Table 5:** Number of articles retrieved for each of the selected infections

Disease	Number of published articles (%)* N = 5606
HIV/AIDS	1537 (27.4)
Coronavirus (SARS, MERS, COVID-19)	1316 (23.5)
Influenza (flu, avian, swine)	692 (12.3)
Malaria	664 (11.8)
Tuberculosis	492 (8.8)
Dengue	302 (5.4)
Ebola	134 (2.4)
Measles	113 (2.0)
Cholera	112 (2.0)
Zika	83 (1.5)
Rabies	57 (1.0)
Pneumonia	46 (0.8)
Polio	34 (0.6)
chikungunya	34 (0.6)
Syphilis	31 (0.6)
Plaque	24 (0.4)
Smallpox	20 (0.4)
Yellow fever	15 (0.3)
Hantaviruses	15 (0.3)

*The total percentage might exceed 100% due to the presence of a certain number of articles discussing more than one disease

### Citation analysis

The retrieved documents received 155,146 citations, a mean of 27.7, and an *h*-index of 158. Table 6 lists the top 10 cited articles [38–47]. The top 10 cited articles included four about COVID-19, four about influenza, and two about HIV. The top 10 cited articles were published in prestigious journals such as *The Lancet, Nature, Science*, and *PNAS*.

**Table 6:** Top ten cited articles on mathematical modeling for the transmission and control of the selected infectious diseases

Rank	Title	Year	Source title	Cited by
1	Universal voluntary HIV testing with immediate antiretroviral therapy as a strategy for elimination of HIV transmission: a mathematical model	2009	*The Lancet*	1471
2	Strategies for mitigating an influenza pandemic	2006	*Nature*	1436
3	Identification and characterization of transmitted and early founder virus envelopes in primary HIV-1 infection	2008	*Proceedings of the National Academy of Sciences of the United States of America*	1405
4	Strategies for containing an emerging influenza pandemic in Southeast Asia	2005	*Nature*	1340
5	The effect of travel restrictions on the spread of the 2019 novel coronavirus (COVID-19) outbreak	2020	*Science*	1319
6	Feasibility of controlling COVID-19 outbreaks by isolation of cases and contacts	2020	*The Lancet Global Health*	1129
7	Early dynamics of transmission and control of COVID-19: a mathematical modelling study	2020	*The Lancet Infectious Diseases*	1044
8	Containing pandemic influenza at the source	2005	*Science*	921
9	The effect of control strategies to reduce social mixing on outcomes of the COVID-19 epidemic in Wuhan, China: a modelling study	2020	*The Lancet Public Health*	917
10	Mitigation strategies for pandemic influenza in the United States	2006	*Proceedings of the National Academy of Sciences of the United States of America*	776

## Discussion

The current study was a bibliometric analysis of literature on mathematical modeling of transmission and control of a group of known infectious diseases. Implementing mathematical and statistical sciences in understanding the transmission, and control of infectious diseases became an important and commonly used topic in the past several months due to the emergence of a new coronavirus, COVID-19. Understanding infectious disease dynamics is an important step in understanding the spread and methods of control of infectious disease outbreaks. Therefore, mathematical modeling and its application in medicine in general and in the field of infectious diseases, in particular, is important to all countries and nations. Recently, national and global policies of sustainable cross-border closure during COVID-19 were based on mathematical modeling of the transmission of the virus [48]. Mathematical modeling was critical in developing policies related to social distancing, mask-wearing, and hand hygiene during COVID-19 crisis [49–51]. Mathematical modeling was also used to combat measles by implementing vaccination policies for certain categories of people [52–54]. Mathematical modeling was also suggested for drawing policies to decrease the progression of antimicrobial resistance [55, 56].

The current study indicated that the number of publications in this field witnessed a gradual increase in the past decade and a sharp increase in the last two years. The gradual increase in the number of publications could be attributed to several factors. First, several infectious disease outbreaks have affected the world since the year 2000 including influenza outbreaks, Ebola, Severe Acute Respiratory Syndrome (SARS), Zika, and many others. Second, the call for papers made by several journal editors for submission of papers on mathematical modeling of infectious diseases. The *PLOS* series and Hindawi publishers published a special collection of articles on this topic lately. Third, the vast development of vaccines for many infectious diseases enabled health authorities to carry out mass vaccination/immunization and could change the pattern of infectious disease dynamics [57, 58]. Fourth, the recent advancement of technology, computer programing enabled mathematicians to use complex equations to predict future patterns based on certain early epidemiologic data [59, 60]. Fifth, the growth of population and the ease of travel made humans get closer to each other and increase the risk of the rapid spread of infectious diseases [61]. It was important and necessary given these new factors to develop models to predict and control infectious disease transmission and spread. Sixth, the application of mathematical modeling on both acute infectious diseases and chronic infectious diseases such as HIV/AIDS. Finally, the introduction and use of mathematical modeling in health fields in general enhanced the use of these methods on infectious diseases as well [62, 63]. The sharp increase in the number of publications in the last two years was due to the spread of COVID-19.

The current study showed the dominance of high-income countries with relatively poor contribution and inadequate collaboration with low- and middle-income countries. This is due to several factors. The low- and middle-income countries might lack experts and experience in this field despite that elimination and prevention of infectious diseases is a top priority for all countries. In this regard, low- and middle-income countries may lack experts in the field of mathematical/statistical sciences or lack collaboration with experts in the field of epidemiology and mathematical sciences. It is also possible that health policymakers in low- and middle-income countries are not aware of the importance and usefulness of this field in understanding, controlling, and prevention of infectious disease dynamics. High-income countries have experts, expertise, technology, and funding to carry out studies in this field. This was also evident in other medical fields where high-income countries ranked top and dominated literature in terms of the number of publications [16, 17, 24, 64]. The dominance of high-income countries in this field was reflected in active authors and institutions where authors and institutions in the UK and the USA dominated the list of active authors and active institutions.

International research collaboration plays an important role in improving quality research, research productivity, and academic impact [65, 66]. In the current study, international research collaboration was seen between researchers in high-income countries or those with a common language or culture. This was evident in the research collaboration map where countries with a high extent of research collaboration existed nearby with thick connecting lines. However, poor research collaboration was seen between high-income and low- and middle-income countries. This poor research collaboration is considered a barrier to the social construction and evolution of new scientific fields such as the mathematical modeling of infectious diseases [67]. Inadequate international research collaboration in this field is a disadvantage for all countries because national health security in low- and middle-income countries can influence global health security. Furthermore, international research collaboration has a positive impact on research output, visibility, and citations [68, 69]. The research output from individual countries is reflected in regional research output. The region of the Americas ranked first and the Eastern Mediterranean region ranked last. The Eastern Mediterranean region has been under the influence of various serious infectious disease outbreaks such as Middle East respiratory syndrome coronavirus (MERS-CoV) [70, 71]. Furthermore, the internal conflicts and the collapse of the health systems in many countries in the Eastern Mediterranean region have generated several outbreaks of infectious diseases among refugees and displaced people. For example, cholera in Yemen and poliomyelitis in Syria [72, 73]. In contrast to the Eastern Mediterranean region, the African region showed a considerable contribution to the retrieved literature. This was not surprising given that Africa is the region for millions of HIV patients, malaria, filariasis, tuberculosis, viral hepatitis, and many other neglected tropical diseases [74]. The international aid to African countries had played a positive role in joint research in fighting common infectious diseases through investigating and developing mathematical models to eliminate such diseases.

The current study showed that the retrieved literature had a relatively lower *h*-index value than other topics. For example, the *h*-index for the literature on emerging pathogens was 173 [16]. However, the *h*-index for the retrieved literature was relatively higher than the literature on parasitic skin diseases or anti-malarial drug resistance [64, 75]. Many factors could affect the number of citations per article including the subject area, the number of authors, the reputation of the journal, and others [76]. Mathematical modeling in medicine is relatively a new concept to most researchers in the health field. However, the application of mathematical modeling on important topics such as HIV/AIDS, malaria, and disease outbreaks attracted a lot of citations since these infectious diseases are very prevalent and have no effective and safe vaccine yet. A final point regarding the citation was the finding that four articles on COVID-19 received a high number of citations and ranked among the top 10 cited articles. The COVID-19 pandemic received global scientific attention because of its rapid spread and potentially fatal outcome.

The current study showed that HIV/AIDS, coronaviruses, influenza, malaria, and tuberculosis were the most frequently researched. These diseases are transmitted by a variety of methods including sexual contacts, airborne, and vector-borne transmission. There are new topics related to HIV/AIDS that attracted the attention of researchers. Such new ideas included HIV-self testing, pre-, and post-prophylaxis therapy, and the introduction of new effective therapy. These new interventional strategies were tested using mathematical modeling for the potential eradication of HIV to meet sustainable development goals [77–79]. For malaria, mathematical modeling was important to estimate the role of climate change, insecticide-treated nets, and new anti-malarial drugs on the transmission dynamics of malaria [80, 81]. Concerning influenza, two serious outbreaks affected most countries in the world. In 2003, the re-emergence of avian flu A(H5N1) and the 2009 “Swine flu” (H1N1) pandemic caused severe illness in previously healthy adults and spread rapidly causing death in hundreds of thousands of people [82]. The fifth major infectious disease in the retrieved literature was tuberculosis which is one of the top 10 causes of death and the leading cause from a single infectious agent (above HIV/AIDS) [83]. In 2018, the 30 high TB burden countries accounted for 87% of new TB cases. Eight countries account for two-thirds of the total, with India leading the count, followed by, China, Indonesia, the Philippines, Pakistan, Nigeria, Bangladesh, and South Africa [84]. Three of these countries were listed in the top ten active list. The findings in the current study regarding the main pathogen/infectious diseases were similar to the findings of mathematical modeling for antimicrobial resistance where most articles were about HIV/AIDS, influenza, malaria, tuberculosis, and methicillin-resistant *staphylococcus aureus* [56]. This is another reason for the dominance of these diseases in the retrieved documents. The development of resistance to antiretroviral drugs or anti-malarial or anti-tuberculosis is expected to have serious public health consequences.

The current study is the first of its kind and presented baseline data on research activity on mathematical modeling on transmission and control of infectious diseases. However, the current study has a few limitations. First, the use of Scopus is not the perfect approach since many journals in different world regions are not indexed in Scopus. However, Scopus remains the best available database for analysis of research activity and finding research hotspots on a certain topic. Second, the search query was comprehensive, but missing a few relevant keywords remains a possibility. Not all infections have been listed in the search query. However, the author did his best to include infections with a known history of serious outbreaks. Third, the lists of active key players were retrieved from Scopus directly. Sometimes, due to the presence of several different names or spelling, the research output of certain authors, countries, or institutions is under-estimated in the Scopus database.

## Conclusion

In recent years, applications of mathematics in infectious disease have shown remarkably growing trends and have been used most commonly in HIV/AIDS, Coronavirus disease, malaria, influenza, and tuberculosis. High-income countries dominated the field and showed poor international research collaboration with low- and middle-income countries. Low- and middle-income countries need to build their expertise in this field through training and collaboration with active institutions and authors. Health authorities also need to fund and invest in building knowledge and expertise in this field to be able to understand and prevent the spread of infectious diseases based on scenarios available in each country. Finally, the current study is not comprehensive of all infection-related conditions. Therefore, the current study could be an initial step for future studies in this field. The author recommends doing similar studies for specific pathogens considered a top priority by the WHO due to their rapid emergence of resistance in certain pathogens.

## Data Availability

all data presented in this manuscript are available on Scopus database using the search query listed in the methodology section.
